# Divergent Roles of Central Serotonin in Adult Hippocampal Neurogenesis

**DOI:** 10.3389/fncel.2017.00185

**Published:** 2017-06-30

**Authors:** Ning-Ning Song, Ying Huang, Xin Yu, Bing Lang, Yu-Qiang Ding, Lei Zhang

**Affiliations:** ^1^Key Laboratory of Arrhythmias, Ministry of Education, East Hospital, Tongji University School of MedicineShanghai, China; ^2^Department of Anatomy and Neurobiology, Tongji University School of MedicineShanghai, China; ^3^Mental Health Institute of the Second Xiangya Hospital, National Clinical Research Center on Mental Disorders, National Technology Institute on Mental Disorders, Key Laboratory of Psychiatry and Mental Health of Hunan Province, Central South UniversityChangsha, China

**Keywords:** adult hippocampal neurogenesis, 5-HT, 5-HT receptors, conventional knockout mouse, conditional knockout mouse

## Abstract

The central serotonin (5-HT) system is the main target of selective serotonin reuptake inhibitors (SSRIs), the first-line antidepressants widely used in current general practice. One of the prominent features of chronic SSRI treatment in rodents is the enhanced adult neurogenesis in the hippocampus, which has been proposed to contribute to antidepressant effects. Therefore, tremendous effort has been made to decipher how central 5-HT regulates adult hippocampal neurogenesis. In this paper, we review how changes in the central serotonergic system alter adult hippocampal neurogenesis. We focus on data obtained from three categories of genetically engineered mouse models: (1) mice with altered central 5-HT levels from embryonic stages, (2) mice with deletion of 5-HT receptors from embryonic stages, and (3) mice with altered central 5-HT system exclusively in adulthood. These recent findings provide unique insights to interpret the multifaceted roles of central 5-HT on adult hippocampal neurogenesis and its associated effects on depression.

## Introduction

Neurons in the central nervous system are produced from neural stem/progenitor cells (NSPCs) at embryonic or early postnatal stages in a process called neurogenesis. In the adult brain, this new-neuron production machinery is limited to two regions: the subgranular zone (SGZ) of the hippocampus, which generates glutamatergic granule cells of dentate gyrus (DG), and the subventricular zone (SVZ) lining the lateral ventricles, which produces GABAergic/dopaminergic cells committed to the olfactory bulb (Vadodaria and Gage, 2014). To date, substantial progress has been made to elucidate the mechanisms underlying NSPC activity and subsequent neuronal differentiation and integration into the existing neural network. Key effectors in these different developmental steps include cell intrinsic and extrinsic factors, such as transcriptional factors, morphogens, growth factors, neurotransmitters, and network activity.

Among neurotransmitters, serotonin (5-HT) has attracted the most interest because of the enhancement of adult hippocampal neurogenesis induced by selective serotonin reuptake inhibitors (SSRIs), which might contribute to their antidepressant effects (Sahay and Hen, 2007; Vaidya et al., 2007). SSRIs primarily target the 5-HT system rather than other neurotransmitter systems. Both acute and chronic administration of SSRIs increases the synaptic (extracellular) serotonin concentration by several folds. In the majority of studies of SSRI function in adult hippocampal neurogenesis in rodents, chronic rather than acute administration of SSRIs enhances the proliferation of NSPCs, increases the survival of adult-born neurons, and accelerates the maturation of immature neurons (Malberg et al., 2000; Santarelli et al., 2003; Wang et al., 2008).

Although the higher extracellular level of 5-HT induced by chronic SSRI administration enhances adult neurogenesis, some different conclusions have been drawn from individual genetic mouse models with dysfunctional 5-HT systems. Here, we focus on conclusions from (1) mouse models with altered central 5-HT levels from embryonic stages, (2) mouse models with deletion of 5-HT receptors from embryonic stages, and (3) mouse models with an altered central 5-HT system exclusively in adulthood. We have summarized recent findings to better understand the complicated roles of the central 5-HT system in adult hippocampal neurogenesis.

## Genetic Mouse Models with Altered Central 5-HT Levels from Embryonic Stages

5-HTergic neurons are differentiated from progenitor cells at embryonic day (E) 10.5–11.5, and serotonin is synthesized around E12.5 in mouse brain (Gaspar et al., 2003; Suri et al., 2015). Thus, conventional or “non-inducible” conditional genetic mouse models targeting the genes expressed in 5-HTergic neurons would lead to changes in 5-HT levels from embryonic stages.

### Genetic Mouse Models with Deficiency of Central 5-HT from Embryonic Stages

In brain, tryptophan hydroxylase-2 (Tph2) is the rate-limiting enzyme in the process of central 5-HT synthesis (Zhang et al., 2004), and mouse models with loss or reduction of Tph2 function present defective 5-HT synthesis (Mosienko et al., 2015). Loss or reduction of 5-HT, however, does not affect the survival of 5-HTergic neurons in different Tph2-deficient mouse models (Gutknecht et al., 2008; Alenina et al., 2009; Jia et al., 2014), and therefore, these mice show central 5-HT deficiency rather than 5-HTergic neuronal loss. At young (42 days old) and adult (80 days old) stages, Tph2 conventional KO mice showed normal BrdU-labeled proliferating NSPCs and a baseline level of DCX-labeled immature neurons in the SGZ, but increase of adult neurogenesis induced by exercise is blocked in Tph2 KO mice (Klempin et al., 2013), indicating that exercise-induced adult neurogenesis requires an intact central 5-HT system. Our study confirmed this finding as shown by the normal basal proliferation rate of NSPCs in the DG of Tph2 conditional KO mice (Pet1-Cre; Tph2^flox/flox^; referred to as Tph2^Pet1^ CKO) before aging (Song et al., 2016b). In addition, Tph2-deficient mice do not have increased anxiety-like or depression-like behaviors (Savelieva et al., 2008; Mosienko et al., 2012; Angoa-Perez et al., 2014; Jia et al., 2014). The response to SSRIs is abolished in a subset of Tph2 KO mice (Angoa-Perez et al., 2014), thus showing that the antidepressant effects of SSRIs indeed partially depend on an intact 5-HT system, but central 5-HT deficiency is not a prerequisite in the onset of depression-like behaviors, at least in mice.

Vmat2 transports 5-HT from cytosol into synaptic vesicles (Reimer et al., 1998). By crossing Vmat2^flox/flox^ mice with Sert-Cre mice, Vmat2^sert-cre^ conditional KO (Sert-Cre; Vmat2^flox/flox^) mice can be generated, which leads to Vmat2 deletion in 5-HTergic neurons. In Sert-Cre mice, however, Cre recombinase expression is not limited in 5-HTergic neurons (Narboux-Neme et al., 2008), and this may lead to Vmat2 deletion in other neurons. Loss of Vmat2 in 5-HTergic neurons results in a decrease of 5-HT in brain, but it does not affect the survival of 5-HTergic neurons (Narboux-Neme et al., 2011). Adult Vmat2^sert-cre^ CKO mice show normal proliferation of adult hippocampal progenitors but enhanced survival of newborn neurons (Diaz et al., 2013). Similar to Tph2-deficient mice, Vmat2^sert-cre^ CKO mice do not show increased anxiety-like behaviors, despite the reduced level of 5-HT in the brain (Narboux-Neme et al., 2011).

Lmx1b and Pet1 are important transcriptional factors involved in the differentiation of 5-HTergic neurons. In Lmx1b conditional KO (Pet1-Cre; Lmx1b^flox/flox^, Lmx1b^Pet1^ CKO) mice, almost all 5-HTergic neurons are lost, which irreversibly results in central 5-HT deficiency (Zhao et al., 2006; Dai et al., 2008). We found that Lmx1b^Pet1^ CKO mice also show normal adult neurogenesis at young and adult age (Song et al., 2016b). Like Tph2^Pet1^ CKO mice, depression-like behaviors are normal at the baseline level but anxiety-like behaviors are reduced in Lmx1b^Pet1^ CKO mice (Dai et al., 2008; Jia et al., 2014). Pet1 regulates Tph2 and Sert expression by directly binding to their promoter domains (Hendricks et al., 1999). Genes associated with 5-HTergic neurons are downregulated, and about 80% reduction of central 5-HT level is present in Pet1-deficient mice (Hendricks et al., 2003). Unlike Lmx1b^Pet1^ CKO mice, most of 5-HTergic neurons survive in Pet1 KO mice (Krueger and Deneris, 2008). Similar to Vmat2^sert-cre^ CKO mice, the survival rather than proliferation of adult-born hippocampal neurons is increased in the DG of Pet1 KO mice (Diaz et al., 2013). Overall, it is somewhat unexpected that these genetic mouse models with a deficiency in central 5-HT or loss of 5-HTergic neurons from embryonic stages display normal baseline level of proliferation in adult hippocampal neurogenesis at young and adult age.

### Genetic Mouse Models with Elevated (Extracellular) 5-HT Levels from Embryonic Stages

Sert, the specific 5-HT transporter, is expressed in central 5-HTergic neurons and is responsible for transporting synaptic 5-HT back into 5-HTergic neuronal cytoplasm. In Sert KO mice, the extracellular 5-HT level is increased dramatically (Bengel et al., 1998; Shen et al., 2004). However, the 5-HT concentrations in some brain regions, including brain stem, forebrain, hippocampus, and striatum, are reduced significantly, which may be caused by the reduction of 5-HTergic neurons in Sert KO mice (Lira et al., 2003). A previous study showed that the proliferation of NSPCs in the DG of Sert KO mice is comparable to control mice (Schmitt et al., 2007), but a recent study reported an increased proliferation of NSPCs in Sert KO mice at adult age (Karabeg et al., 2013). In addition, more granule cells in the DG express immediate early genes (Karabeg et al., 2013), which might indicate higher activity of granule neurons in the DG of Sert KO mice.

Serotonin can be degraded by MaoA/B, whose deficiency leads to a higher 5-HT level in the brain (Chen et al., 2004). More proliferating cells in the DG are found in MaoA/B deficient mice (Singh et al., 2013). However, as MaoA/B also degrades other monoamines, dopamine and norepinephrine levels also are increased after deletion of MaoA/B (Chen et al., 2004). Therefore, the phenotype of adult hippocampal neurogenesis cannot be attributed entirely to an increase of the central 5-HT level. **Table 1** provides a summary of findings about adult hippocampal neurogenesis from these genetic mouse models with altered 5-HT levels from embryonic stages.

**Table 1:** Adult hippocampal neurogenesis in genetic mouse models with altered central 5-HT levels from embryonic stages or adulthood.

Adult hippocampal neurogenesis in genetic mouse models with altered central 5-HT levels from embryonic stages							
**Genetic mouse models**	**Central 5-HT**	**Survival of 5-HT^+^ neurons**	**Proliferation of NSPCs**	**Survival of adult-born neurons**	**Neurogenesis induced by exercise or EE**	**Maturation of adult-born neurons**	**References**

Tph2 KO	Almost lost	Normal	Normal in adult, increased in aged	N.D.	Blocked	N.D.	Alenina et al., 2009; Klempin et al., 2013

Tph2^Pet1^ CKO	Almost lost	Normal	Normal in adult, increased in aged	N.D.	N.D.	Enhanced dendritic length of adult-born neurons	Jia et al., 2014; Song et al., 2016b

Vmat2^sert-cre^ CKO	Almost lost	Normal	Normal in adult	Increased	N.D.	N.D.	Narboux-Neme et al., 2011; Diaz et al., 2013

Lmx1b^Pet1^ CKO	Almost lost	Lost almost all 5-HT^+^ neurons	Normal in adult, increased in aged	N.D.	N.D.	N.D.	Zhao et al., 2006; Dai et al., 2008; Song et al., 2016b

Pet1 KO	Reduced about 80%	Almost normal	Normal in adult, N.D. in aged	Increased	N.D.	N.A.	Hendricks et al., 2003; Krueger and Deneris, 2008; Diaz et al., 2013

Sert KO	Tissue 5-HT reduced, extracellular 5-HT increased	Lost about 50% 5-HT^+^ neurons	Normal in adult, increased in aged (Schmitt et al., 2007), Increased in adult (Karabeg et al., 2013)	N.D.	N.D.	N.D.	Bengel et al., 1998; Lira et al., 2003; Shen et al., 2004; Schmitt et al., 2007; Karabeg et al., 2013

MaoA/B double KO	Increased	N.D.	Increased in adult, N.D. in aged	N.D.	N.D.	N.D.	Chen et al., 2004; Singh et al., 2013

**Adult hippocampal neurogenesis in mouse models with altered 5-HT level exclusively from adulthood**							

**Mouse models**	**Central 5-HT**	**Survival of 5-HT^+^ neurons**	**Proliferation of NSPCs**	**Survival of adult-born neurons**	**Neurogenesis induced by exercise or EE**	**Maturation of adult-born neurons**	**References**

hTM-DTA^iPet1^	Almost lost	Lost almost all 5-HT^+^ neurons	Increased	Increased	N.D.	N.D.	Song et al., 2016a

lTM-DTA^iPet1^	Reduction about 50% of Tph2^+^ cells	Lost half of 5-HT^+^ neurons	Normal	N.D.	N.D.	N.D.	Song et al., 2016a

PC/DTR	Almost lost	Lost almost all 5-HT^+^ neurons	Increased	N.D.	N.D.	Enhanced dendritic length of new-born neurons	Jia et al., 2014; Song et al., 2016b

Pet1-CreER^T2^; Tph2^flox/flox^ CKO	Reduction about 80% of Tph2^+^ cells	Normal	Increased	N.D.	N.D.	N.D.	Song et al., 2016a

Sert RNAi	Increased extracellular 5-HT level	N.D.	Increased	N.D.	N.D.	N.D.	Ferres-Coy et al., 2013

*EE, enriched environment; N.D., not determined.*

The decease or increase of central 5-HT levels from the embryonic stages does not simply show opposite effects on adult hippocampal neurogenesis, and most reports have demonstrated that the basal proliferation of adult hippocampal NSPCs is maintained at a normal rate in these genetic mouse models. As described in a review on this topic (Teissier et al., 2017), 5-HT is implicated in regulating brain development, including the establishment of its own axon projection map. Thus, developmental defects are present in the brain of these genetic mouse lines because of the loss of 5-HT, and they are not optimal tools to address the direct roles of central 5-HT in regulating adult neurogenesis.

## Genetic Mouse Models with Deletion of 5-HT Receptors from Embryonic Stages

5-HT is released to synaptic cleft and functions after binding to its receptors. Thus far, 14 types of 5-HT receptors have been identified (Barnes and Sharp, 1999). The molecular and pharmacological features of 5-HT receptors have been reviewed elsewhere (Hoyer et al., 2002) and are not the major point of this review. How these receptors regulate adult neurogenesis is studied widely by acute or chronic administration of their agonists and antagonists. A recent review has summarized the effects of drugs on adult neurogenesis (Alenina and Klempin, 2015). Here, we focus on the data obtained from genetic mouse models of 5-HT receptors. Most of the genetic mouse models are conventional KO mice with total rather than specific deletion of 5-HT receptors.

5-HTR1A acts as both an autoreceptor and a heteroreceptor in mediating 5-HT function. 5-HTR1A inhibits firing of 5-HTergic neurons as an autoreceptor. It is highly expressed in many brain regions, including dorsal raphe nucleus and hippocampus. Most studies showed that the agonists of 5-HTR1A promote proliferation of NSPCs or survival of newborn neurons in adult hippocampal neurogenesis (Alenina and Klempin, 2015). Baseline level of adult neurogenesis is not affected in conventional 5-HTR1A KO mice, although increased proliferation of NSPCs in hippocampus induced by fluoxetine is blocked in these mice (Santarelli et al., 2003). A recent study found that higher proliferation of hippocampal NSPCs in mice housed in enriched environment is blocked in 5-HTR1A KO mice (Rogers et al., 2016). Different region-specific and time-controlled 5-HTR1A-deficient mice have been generated to investigate the function of 5-HTR1A in behaviors associated with mood and so on (Donaldson et al., 2013). More information and a precise conclusion about the function of 5-HTR1A in adult neurogenesis could be obtained from these genetic mouse models. Like 5-HTR1A, 5-HTR1B also acts as both an autoreceptor and a heteroreceptor. To date, no research has been performed to address adult neurogenesis in 5-HTR1B single KO mice. Double KOs of 5-HTR1A and 1B receptors, however, lead to decreased survival of adult-born neurons rather than proliferation of NSPCs in DG (Xia et al., 2012).

Similar to 5-HTR1A, baseline level of proliferation of NSPCs and survival of adult-born neurons is not affected in 5-HTR2B KO mice. However, the increased proliferation and survival induced by chronic fluoxetine administration is blocked in 5-HTR2B KO mice (Diaz et al., 2012). 5-HTR3 is the only ionotropic receptor in the 5-HT receptor family. The baseline level of proliferation of NSPCs and survival of adult-born neurons are not affected in 5-HTR3 KO mice; however, elevated adult neurogenesis and antidepressant-like behaviors induced by exercise is blocked in such mice (Kondo et al., 2015).

For 5-HTR4, its agonist has rapid anxiolytic–antidepressant effects (Mendez-David et al., 2014) and stimulates adult neurogenesis, but enhanced hippocampal neurogenesis is not required for these effects. In 5-HTR4 conventional KO mice, the progenitors and immature neurons are not affected at the baseline level, although the increase of these two types of cells induced by fluoxetine is blocked (Imoto et al., 2015). In this study (Imoto et al., 2015), the authors showed that 5-HTR4 is expressed in mature granule cells rather than in immature neurons in the hippocampus, which indicates an indirect involvement of 5-HTR4 in adult hippocampal neurogenesis. For 5-HTR7 receptor, the proliferation of NSPCs in the hippocampus is not changed in KO mice (Sarkisyan and Hedlund, 2009). Until now, adult neurogenesis has not been addressed in some 5-HT receptor KO mice, such as 5-HTR1D, 1E, 1F, and so on. **Table 2** summarizes the adult neurogenesis in 5-HT receptor conventional KO mice.

**Table 2:** Adult hippocampal neurogenesis in genetic mouse models with deletion of 5-HT receptors from embryonic stages.

Genetic mouse models	Proliferation of NSPCs	Survival of adult-born neurons	Neurogenesis induced by exercise or EE	Neurogenesis induced by SSRIs	References
5-HTR1A KO	Normal	N.D.	Blocked	Blocked	Santarelli et al., 2003; Rogers et al., 2016
5-HTR1A/1B double KO	Normal	Increased	N.D.	N.D.	Xia et al., 2012
5-HTR2B KO	Normal	Normal	N.D.	Blocked	Diaz et al., 2012
5-HTR3 KO	Normal	Normal	Blocked	N.D.	Kondo et al., 2015
5-HTR4 KO	Normal	N.D.	N.D.	Blocked	Imoto et al., 2015
5-HTR7 KO	Normal	N.D.	N.D.	N.D.	Sarkisyan and Hedlund, 2009

*EE, enriched environment; N.D., not determined.*

5-HT receptors may have synergic effects to modulate adult neurogenesis. Additionally, in conventional KO mice with the defined deletion of a 5-HT receptor, the activity of other 5-HT receptors may compensate. Therefore, further study may need to investigate the adult hippocampal neurogenesis in multiple-gene KO mice with deletion of several receptors simultaneously.

Different receptors may play different roles in regulating adult hippocampal neurogenesis: some receptors contribute to the baseline level of adult hippocampal neurogenesis, whereas others are required for excise- or enriched environment-induced hippocampal neurogenesis.

## Genetic Mouse Models with Altered 5-HT System Exclusively in Adulthood

In conventional or non-inducible KO mice, a change in the 5-HT level or 5-HT receptors begins in the embryonic stages, and as mentioned, developmental defects or possible compensations present in these mice interfere with conclusions obtained from these mice. Therefore, time-controlled inducible conditional KO mice with normally developed brains are required to address the roles of 5-HT system in adult hippocampal neurogenesis. In our recent work, time-controlled inducible genetic mouse models are generated with central 5-HT deficiency exclusively from adulthood. Three mouse models with time-controlled 5-HT deficiency have been used: (1) Pet1-Cre; Rosa26-DTR (diphtheria toxin receptor) mice obtained by crossing Pet1-Cre with Rosa26-DTR mice in which 5-HTergic neurons are depleted after diphtheria toxin (DT) is injected (Jia et al., 2014); (2) Pet1-CreER^T2^; Rosa26-DTA mice generated by crossing Pet1-CreER^T2^ with Rosa26-DTA mice in which 5-HTergic neurons would be deleted after tamoxifen is injected; and (3) Pet1-CreER^T2^; Tph2^flox/flox^ CKO mice are generated by crossing Pet1-CreER^T2^ with Tph2^flox/flox^ mice in which central 5-HT synthesis are blocked after tamoxifen is injected (Song et al., 2016a).

In these three mouse models, central 5-HT levels are reduced specifically after administration of DT or tamoxifen in adulthood, and these levels also can be controlled by the drug dosage (Song et al., 2016a). We found that adult hippocampal neurogenesis is enhanced as shown by increased proliferation of NSPCs and survival of newborn neurons in the DG of DT-injected Pet1-Cre; Rosa26-DTR mice (PC/DTR mice) and high-dose tamoxifen-administrated adult Pet1-CreER^T2^; Rosa26-DTA mice (hTM-DTA^iPet1^ mice) with a loss of about 95% of central 5-HTergic neurons (Jia et al., 2014; Song et al., 2016a). In contrast, the loss of half of the central 5-HTergic neurons in adult Pet1-CreER^T2^; Rosa26-DTA mice (lTM-DTA^iPet1^ mice) with a low dose of tamoxifen administration does not lead to significant alterations of adult hippocampal neurogenesis (Song et al., 2016a). In Pet1-CreER^T2^; Tph2^flox/flox^ CKO mice with tamoxifen administration in adulthood, however, 5-HT level are estimated in 20% of controls, and the progenitors and immature neurons in the DG also are increased but to a lesser extent than in hTM-DTA^iPet1^ mice (Song et al., 2016a). Thus, lowering central 5-HT to an extremely low level also can enhance adult hippocampal neurogenesis in mice with normally developed brains. This result, in part, is consistent with a previous study demonstrating that survival rather than proliferation is enhanced in mice chronically treated with para-chlorophenylalanine (PCPA), an inhibitor of 5-HT synthesis (Diaz et al., 2013). In addition, we also found that the dendritic length of adult-born neurons is increased in DT-treated adult PC/DTR mice (Song et al., 2016b). Combined with data from administration of SSRIs, we conclude that in a normally developed brain, hippocampal neurogenesis is enhanced in two opposite conditions of central 5-HT levels, extremely low or high. It is possible that an extremely low or high central 5-HT level activates different 5-HT receptor combinations, which results in similar phenotypes of adult hippocampal neurogenesis.

Chronic administration of SSRIs, which inhibits the function of Sert and therefore augments extracellular 5-HT level, promotes adult hippocampal neurogenesis. Until now, however, there is no inducible Sert KO mouse available with Sert-deficiency exclusively in adulthood. In mice with downregulation of Sert by RNA interference in adulthood, the proliferation of NSPCs and the number of immature neurons are increased in the DG (Ferres-Coy et al., 2013). Because of the limitations of *in vivo* RNAi, time-controlled Sert conditional KO mice by crossing Sert-floxed mice (Chen et al., 2015) with Pet1-CreER^T2^ would be helpful to address the effect of higher extracellular 5-HT on adult hippocampal neurogenesis. **Table 1** provides a summary of the mouse models discussed here.

Collectively, central 5-HT regulates adult neurogenesis in different ways, as shown by data from mice with dysfunctional 5-HT from embryonic stages or adulthood. The function of SSRIs on depression- and anxiety-like behaviors show age dependence (Ansorge et al., 2008; Homberg et al., 2010; Olivier et al., 2011), which may be caused by the broad and transient expression of Sert in the developing brain (Narboux-Neme et al., 2008; Homberg et al., 2010). Increased neurogenesis by SSRIs, however, is reduced or blocked in aged mouse models (Couillard-Despres et al., 2009), suggesting that aging is a key factor affecting adult hippocampal neurogenesis induced by experimental manipulations.

## Late-Onset Elevated Neurogenesis in Genetic Mouse Models with Altered Central 5-HT Levels from Embryonic Stages

In Tph2 conventional KO mice, the proliferation of NSPCs in the DG is normal at young and adult age, but it is increased at aged stage (Klempin et al., 2013). Similar phenotypes are also present in Tph2 and Lmx1b conditional KO mice in which these genes have been deleted in the embryonic stage (Song et al., 2016b). Additionally, an increase in the dendritic length of newborn neurons is present in aged rather than young adult Tph2 and Lmx1b CKO mice (Song et al., 2016b). As mentioned, this late-onset phenotype of enhanced proliferation of NSPCs also is observed in Sert KO mice (Schmitt et al., 2007). It is still unclear why these mouse lines with altered 5-HT systems from the embryonic stage have normal hippocampal neurogenesis at young and adult age but show enhanced neurogenesis when aged.

Up- or downregulation of 5-HT system function does not simply cause opposite effects on adult hippocampal neurogenesis. 5-HT may function in different ways in regulating adult neurogenesis with altered 5-HT system from embryonic stages or from adulthood. Future studies are needed to explore the multifaceted roles of central 5-HT on adult hippocampal neurogenesis.

## Author Contributions

N-NS, YH, XY, BL, Y-QD, and LZ wrote the paper.

## Conflict of Interest Statement

The authors declare that the research was conducted in the absence of any commercial or financial relationships that could be construed as a potential conflict of interest.
